# Effect of adjuvant chemoradiotherapy on overall survival of gastric cancer patients submitted to D2 lymphadenectomy

**DOI:** 10.1007/s10120-012-0171-4

**Published:** 2012-06-29

**Authors:** Alexandre A. A. Jácome, Durval R. Wohnrath, Cristovam Scapulatempo Neto, José Humberto T. G. Fregnani, Ana Luiza Quinto, Antônio T. T. Oliveira, Vinícius L. Vazquez, Gilberto Fava, Edson Z. Martinez, José S. Santos

**Affiliations:** 1 Department of Gastrointestinal Medical Oncology, Barretos Cancer Hospital, Str Antenor Duarte Villela 1331, Barretos, SP 14784-400 Brazil; 2 Department of Gastrointestinal Surgical Oncology, Barretos Cancer Hospital, Str Antenor Duarte Villela 1331, Barretos, SP 14784-400 Brazil; 3 Department of Pathology, Barretos Cancer Hospital, Str Antenor Duarte Villela, 1331, Barretos, SP 14784-400 Brazil; 4 Center for Researcher Support, Barretos Cancer Hospital, Str Antenor Duarte Villela, 1331, Barretos, SP 14784-400 Brazil; 5 Department of Radiation Oncology, Barretos Cancer Hospital, Str Antenor Duarte Villela, 1331, Barretos, SP 14784-400 Brazil; 6 Department of Social Medicine, University of São Paulo at Ribeirão Preto School of Medicine, Av Bandeirantes, 3900, 2nd Floor, Ribeirão Preto, SP 14049-900 Brazil; 7 Department of Surgery and Anatomy, University of São Paulo at Ribeirão Preto School of Medicine, Av Bandeirantes, 3900, 9th Floor, Ribeirão Preto, SP 14049-900 Brazil

**Keywords:** Stomach neoplasms, Adjuvant chemotherapy, Adjuvant radiotherapy, Lymph node excision, Survival

## Abstract

**Background:**

Adjuvant chemoradiotherapy (CRT) is the standard treatment in Western countries for gastric cancer patients submitted to curative resection. However, the role of adjuvant CRT in gastric cancer treated with D2 lymphadenectomy has not been well defined.

**Methods:**

We conducted a retrospective study in patients with stage II to IV gastric adenocarcinoma with no distant metastases, who underwent curative resection with D2 lymphadenectomy between January 2002 and December 2007. The present study compared the 3-year overall survival of two treatments (adjuvant CRT according to the INT 0116 trial versus resection alone). Survival curves were estimated by the Kaplan–Meier method and compared with a log-rank test. Multivariate analysis of prognostic factors was performed by the Cox proportional hazards model.

**Results:**

A total of 185 patients were included, 104 patients (56 %) received adjuvant CRT and 81 received resection alone. The 3-year overall survival was 64.4 % in the CRT group and 61.7 % in the resection-alone group (*p*: 0.415). However, according to the Cox proportional hazards model, adjuvant CRT was a prognostic factor for 3-year overall survival (hazard ratio [HR] 0.46, 95 % confidence interval [CI] 0.26–0.82, *p*: 0.008).

**Conclusions:**

In the present study, adjuvant CRT was associated with a lower risk of death over a 3-year period in gastric cancer patients treated with D2 lymphadenectomy.

## Introduction

Gastric cancer is a malignant neoplasm with the fourth-highest incidence worldwide, represents the second-greatest cause of cancer-related deaths, and frequently affects the populations of Latin America, Eastern Europe, China, and Japan [1, 2].

Gastric cancer that is limited to the stomach and to regional lymph nodes is potentially curable by surgical resection. However, about 20–40 % of all patients submitted to resection with no further treatment will suffer a relapse of the disease within 2 years [3, 4]. Once a relapse occurs, no curative treatment is available and the median survival is about 6 months [3].

In view of these unfavorable outcomes of recurrent gastric cancer, adjuvant treatments have been tested during the past decade. In 2001, based on the results of the INT 0116 trial, adjuvant chemoradiotherapy with 5-fluorouracil started to be widely accepted in Western countries by demonstrating a significant benefit in overall survival [5]. However, the results of the trial were contested because only 10 % of the population studied had been submitted to D2 lymphadenectomy, which is considered to reduce the risk of locoregional recurrence and the risk of death related to gastric cancer [5, 6].

The presence of residual disease in nonresected lymph nodes, which can be measured with a quantitative estimator denoted as the Maruyama index, is an independent prognostic factor in patients with gastric cancer [7–9]. The addition of radiotherapy to adjuvant therapy can be a useful strategy in patients submitted to more limited dissections, with a higher risk of microscopic locoregional disease. In patients with more extensive dissections, radiotherapy may not be of additional benefit in terms of survival and may actually have a deleterious effect by increasing the toxicity of treatment [5, 10–12].

Recent data support the hypothesis that patients with gastric cancer benefit from adjuvant chemotherapy [13], but there is still no agreement about the therapeutic regimen to be employed. Perioperative chemotherapy or adjuvant chemotherapy alone has proven to be promising in clinical trials with predominantly D2 lymphadenectomy dissection [14–17].

The objective of the present study was to assess the influence of adjuvant chemoradiotherapy on the overall survival of patients with gastric cancer submitted to D2 lymphadenectomy.

## Patients and methods

This was a retrospective cohort study conducted in patients with a histological diagnosis of stage II to IV gastric or esophagogastric junction (EGJ) adenocarcinoma, with no distant metastases, according to the classification of the American Joint Committee on Cancer 6th edition. These patients were submitted to curative resection and D2 lymphadenectomy at the Barretos Cancer Hospital (Barretos, São Paulo, Brazil) between January 2002 and December 2007.

Surgical treatment consisted of gastrectomy with free margins (R0 resection) and D2 lymphadenectomy, without splenectomy or caudal pancreatectomy. The procedure was performed by a single team of oncology surgeons with training in surgery of the digestive system. The indication for chemoradiotherapy or for resection alone was based on the clinical judgment of the oncology team, which consisted of surgical, medical, and radiation oncologists.

Adjuvant therapy consisted of five cycles of intravenous 5-fluorouracil and leucovorin (425 and 20 mg/m^2^, respectively, daily) for 5 days, every 28 days, concomitant with radiotherapy in cycles two and three, when the dose of 5-fluorouracil was reduced to 400 mg/m^2^, according to the INT 0116 trial [5]. The total radiotherapy dose was 45 Gy in 25 fractions over 5 days of the week and was applied to the tumor bed; areas of anastomosis; and perigastric, celiac, para-aortic, splenic, hepatoduodenal; and pancreatoduodenal lymph nodes. In tumors of the EGJ and of the proximal portions of the stomach, the field was proximally expanded by 3–5 cm to include the distal esophagus area and part of the left hemidiaphragm, as well as regions of paracardiac and paraesophageal lymph nodes. Anteroposterior (AP) and posteroanterior (PA) fields were used in the conventional technique, with a 6-MV linear accelerator or cobalt unit. The cardiac area was protected with blocks and at least 2/3 of one kidney was spared from irradiation.

After the end of treatment, the patients were followed up by anamnesis, physical examination, laboratory tests (blood count, hepatic function and renal function) and a chest radiograph every 3 months during the first 2 years, every 4 months during the third year, and every 6 months starting in the fourth year. Patients were also given an abdominal ultrasound every 4 months during the first 2 years and every 6 months starting in the third year. Computed tomography, nuclear magnetic resonance, and upper digestive endoscopy were performed upon clinical indication.

Recurrence was diagnosed based on clinical examination findings and on the complementary tests performed according to the above schedule, with a description of all sites diagnosed during the follow up. Recurrence at the surgical site, in the anastomosis, and in the regional lymph nodes was considered to be locoregional. Peritoneal implants or ascites with no other evident cause were considered to be signs of peritoneal recurrence. Systemic recurrence was considered if distant metastases appeared, including retroperitoneal lymph nodes. Patients with recurrences were evaluated for palliative chemotherapy.

The study compared the 3-year overall survival, as well as the rates and sites of recurrence, in the group of patients submitted to gastrectomy and D2 lymphadenectomy alone (GL) with these parameters in the group of patients submitted to adjuvant chemoradiotherapy (GL + CRT). Finally, the prognostic factors for survival were identified.

The categorical variables in the two groups were compared by the χ^2^ test and the continuous variables were compared by the Mann–Whitney test. Overall survival was defined as the time, in months, elapsed from the date of surgery to the date of death from any cause. The 3-year overall survival was estimated by the Kaplan–Meier method, and the survival curves were compared by the log-rank test. The patients lost to follow up were censored on the date of last contact with the center. Surviving patients were censored within 36 months for the analysis of overall survival. Prognostic factors were assessed by multivariate analysis using the Cox proportional hazards model, and *p* values of less than 0.05 were considered to indicate statistical significance. The analyses were performed with SPSS 13.0 software (SPSS, Chicago, IL, USA).

The study was approved by the ethics committee of the participating center.

## Results

Of the 185 patients selected, 113 (61 %) were males. The median age was 61 years. The median follow up was 30.8 months. Gastric tumors represented 85 % of the cases, and the remaining tumors were those of the EGJ. All patients underwent R0 resection and D2 lymphadenectomy. Nine patients (4.6 %) died during the postoperative period and were not included in the analysis.

A total of 104 (56 %) patients were submitted to adjuvant chemoradiotherapy, and 81 were treated with resection alone. GL + CRT patients were younger and presented more advanced nodal status and TNM stage than GL patients. The characteristics of the groups are listed in Table 1. The rates and sites of recurrence and the frequency of locoregional, peritoneal, and systemic recurrence are listed in Table 2.

**Table 1:** Characteristics of the patients

Characteristics	GL + CRT	GL	*p*
	No. (%)	No. (%)	
Total	104 (100)	81 (100)	
Male sex	61 (59)	52 (64)	0.443
Median age, years (range)	59 (30–81)	68 (27–88)	0.005
Tumor location			
Stomach	88 (85)	68 (84)	0.455
EGJ	16 (15)	13 (16)	
Surgery performed			
Total gastrectomy	56 (53)	32 (40)	0.244
Subtotal gastrectomy	41 (40)	39 (48)	
Esophagogastrectomy	4 (4)	5 (6)	
Resection of the gastric stump	3 (3)	5 (6)	
Median number of dissected lymph nodes (range)	22 (12–52)	17 (12–66)	0.716
Laurén histology			
Intestinal-type	60 (58)	48 (66)	0.391
Diffuse-type	38 (37)	20 (27)	
Mixed-type	5 (5)	4 (6)	
Undifferentiated	0 (0)	1 (1)	
Tumor depth			
T1	0 (0)	1 (1)	0.678
T2	14 (13)	9 (11)	
T3	83 (80)	65 (80)	
T4	7 (7)	6 (8)	
Nodal status			
N0	21 (20)	30 (37)	0.005
N1	44 (42)	38 (47)	
N2	29 (28)	11 (13)	
N3	10 (10)	2 (3)	
TNM Stage			
II	24 (23)	39 (48)	0.004
IIIA	46 (44)	26 (32)	
IIIB	21 (20)	8 (10)	
IV M0	13 (13)	8 (10)	

*EGJ* esophagogastric junction, *CRT* chemoradiotherapy, *GL* gastrectomy and D2 lymphadenectomy alone

**Table 2:** Overall survival and recurrence rates

Variable	GL + CRT	GL	*p*
3-Year overall survival (%)	64.4	61.7	0.415
Median follow up (months)	31.61	27.37	0.412
Locoregional recurrence (%)	8.9	3.7	0.159
Peritoneal recurrence (%)	13.9	6.2	0.092
Systemic recurrence (%)	17.8	16	0.752

**Fig. 1:**
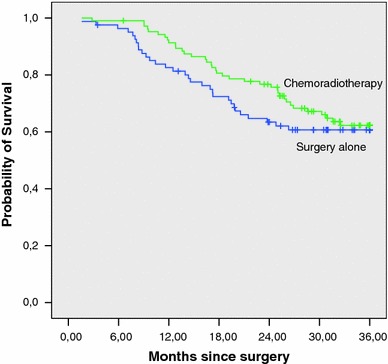
Kaplan–Meier estimation of overall survival

During the follow-up period, 68 deaths occurred, 37 in GL + CRT patients and 31 in GL patients. The 3-year overall survival rate was 63.4 %. Median survival was not reached. The 3-year overall survival rate was 64.4 % for GL + CRT and 61.7 % for GL (*p*: 0.415) (Table 2; Fig. 1).

The Cox proportional hazards model adjusted for age, gender, depth of invasion, nodal status, TNM stage, tumor location, Laurén histology, and adjuvant treatment revealed that only TNM stage and adjuvant treatment had influence on overall survival (Table 3). The patients treated with chemoradiotherapy had a 54 % reduction of the risk of death within 3 years (hazard ratio [HR] 0.46 95 % confidence interval [CI] 0.26–0.82; *p*: 0.008). The HR differed according to stage of disease (Table 4). Patients with no information about Laurén histology (11 patients) were excluded from the multivariate analysis. Only one treatment-related death occurred among GL + CRT patients. The administration of palliative chemotherapy was also similar for both groups, i.e., 16.5 % in GL + CRT and 11.3 % in GL (*p*: 0.313).

**Table 3:** Multivariate analysis of the prognostic factors for 3-year overall survival

Variable	*n* ^a^	Hazard ratio (HR)	95 % Confidence interval (CI)	*p*
Age				
≤60 years	78	–	–	–
>60 years	96	1.37	0.78–2.42	0.267
Gender				
Male	102	–	–	–
Female	72	1.00	0.60–1.66	0.976
Depth of invasion				
Serosa-negative	23	–	–	–
Serosa-positive	151	1.21	0.34–4.24	0.759
Nodal status				
Node-negative	45	–	–	–
Node-positive	129	1.49	0.31–6.98	0.610
TNM stage				
II	57	–	–	–
IIIA	68	1.69	0.38–7.39	0.484
IIIB	29	4.05	0.79–20.57	0.091
IV M0	20	5.50	1.08–27.93	0.040
Location				
Stomach	147	–	–	–
EGJ	27	1.76	0.90–3.44	0.094
Laurén histology				
Intestinal-type	107	–	–	–
Diffuse-type	59	1.29	0.75–2.23	0.354
Mixed-type	8	0.73	0.21–2.44	0.612
Adjuvant therapy				
Resection alone	72	–	–	–
Chemoradiotherapy	102	0.46	0.26–0.82	0.008

^a^A total of 174 patients and 66 events (deaths) were considered in the multivariate analysis

**Table 4:** Estimation of effect of adjuvant CRT on 3-year overall survival stratified by TNM stage

	Stage II	Stage III	Stage IV M0
*n*	63	101	21^a^
HR	0.53	0.70	0.25
95 % CI	0.14–1.96	0.38–1.31	0.08–0.78

^a^A total of 20 patients with stage IV M0 were considered in the analysis, because one patient was censored before the first event

## Discussion

The indication of adjuvant chemoradiotherapy for patients submitted to curative gastrectomy and D2 lymphadenectomy has been debated over the past few years. A retrospective study involving Eastern patients exclusively operated on with D2 lymphadenectomy showed that adjuvant treatment with combined therapy reduced the risks of recurrence and death by 20 % [18]. The ARTIST [17] and CLASSIC [16] trials recently raised the question of the benefit of adjuvant chemoradiotherapy and chemotherapy in Eastern patients submitted to D2 lymphadenectomy. On the other hand, there are no studies that have evaluated the effect of adjuvant chemoradiotherapy according to the pivotal INT 0116 trial in a Western population who have undergone D2 lymphadenectomy exclusively.

In the present study, the patients were evaluated for eligibility for adjuvant treatment after surgery and there was the possibility that postoperative complications (i.e., poor performance status, slow postoperative recovery, poor nutritional status) influenced the decision of the choice of adjuvant therapy. The limitations of this study could explain the heterogeneity of the groups in relation to age, TNM, and node stages, but the use of the regression model adjusted for well-recognized prognostic factors aimed to minimize the influence of these factors on the prognostic value of adjuvant therapy.

Based on the analysis by the regression model with a meaningful hazard ratio (HR), it was suggested that chemoradiotherapy was associated with a lower risk of death within 3 years, with a reduction of 54 %. There was a different benefit derived from adjuvant therapy according to disease stage (Table 4). Patients with stage IV disease with no distant metastases derived the greater benefit from the combined modality therapy, contributing to the hypothesis that patients with more advanced nodal disease could benefit from more intensive local therapy. It is possible that the expressive HR of 0.25 in this subgroup of patients affected the conclusions in relation to the whole population. It is also important to emphasize that such a model-based analysis imposes limitations on conclusions about reduction of the risk of events when compared with design-based analysis, which is usually performed in randomized clinical trials.

The 3-year overall survival of 61.7 % in our patients with gastrectomy and D2 lymphadenectomy alone (GL) is a satisfactory outcome, although inferior to the finding of the ACTS-GS Group trial in a patient population with D2 or D3 lymphadenectomy, which recorded a 3-year overall survival rate of 70.1 % [15]. In the present study, the classification of lymphadenectomy was done on the basis of operative reports; however, there was a wide variation in the number of dissected lymph nodes, with at least 12 in both groups, which is low for D2 resection. This observation, coupled with the presence of a higher percentage of locally advanced disease in the sample studied could explain the 3-year overall survival difference observed between the resection-alone arm of the ACTS-GC Group trial [15] and the similar arm in the present study.

The survival outcome of our group submitted to adjuvant therapy was 64.4 % and this also can be considered satisfactory. In the INT 0116 and ACTS-GS Group trials, the 3-year survival rates were 50 and 80.1 %, respectively [5, 15]. The population investigated in the latter trial presented an earlier stage of disease, with 49.9 % of the patients in stages III and IV with no distant metastases compared with 77 % of the patients in these stages in the present study.

In summary, chemoradiotherapy based on 5-fluorouracil seems to be an effective adjuvant therapy in patients with gastric and EGJ adenocarcinoma submitted to D2 lymphadenectomy, and it will probably continue to be used as one of the options of adjuvant therapy. Through the lessons learned from breast and colon cancers, in which benefits derived from adjuvant therapy have been noted to differ according to the presence of prognostic and predictive factors, and in consideration of the findings of the ARTIST [17] and ToGA [19] trials, maybe it would be interesting to examine carefully the design of more personalized randomized trials for adjuvant therapy in gastric cancer that take into account tailored therapies.
